# Patient Use of a Comment Function in Electronic Health Records: Mixed Methods Case Study

**DOI:** 10.2196/98359

**Published:** 2026-10-20

**Authors:** Josefin Hagström, Anna Kharko, Maria Hägglund

**Affiliations:** 1 Department of Women's and Children's Health Uppsala University Uppsala Sweden; 2 Centre for Primary Care and Health Services Research University of Manchester Manchester United Kingdom; 3 MedTech Science & Innovation Centre Uppsala University Hospital Uppsala Sweden

**Keywords:** patient-accessible electronic health record, PAEHR, patient portal, patient input, communication, case study, artificial intelligence, AI

## Abstract

**Background:**

Patient access to their electronic health records (EHRs) could improve care and reduce errors by enabling patient contribution to documentation. However, interactive functions that support such engagement remain underresearched.

**Objective:**

The purpose of this study was to analyze patients’ use of the free-text comment function in the Swedish national patient-accessible EHR.

**Methods:**

Comments made by patients between 2015 and 2021 for health care professionals to view were retrieved from the patient-accessible EHR administration service in Sweden. The number and length of comments were analyzed using descriptive statistics and chi-square tests, while the content was examined qualitatively using inductive content analysis. In addition, comments were coded for sentiment (positive, neutral, or negative) and note content, and users were classified according to whether they had submitted at least 1 comment referring to mental health care.

**Results:**

A total of 1670 comments were left by 659 patients, of which 15 had irrelevant content (15/1670, 0.9%; 95% CI 0.4%-1.4%), leaving 1655 comments for analysis. Most comments (948/1655, 57.3%; 95% CI 54.9%-59.7%) were related to matters other than note content, and nearly all had a neutral (920/1655, 55.6%; 95% CI 53.2%-58.0%) or negative (702/1655, 42.4%; 95% CI 40.0%-44.8%) sentiment. Patients who submitted a comment referencing mental health care tended to leave more comments overall (median 7 vs 2 comments per patient), and their comments were more frequently negative than comments written by other patients (313/495, 63.2% vs 389/1160, 33.5%; *P*<.001). The most prevalent categories were care engagement, perceived inaccuracies, complaints, and requests.

**Conclusions:**

Patient engagement with clinical notes has the potential to enhance documentation accuracy and strengthen patient-clinician communication. To realize these benefits safely, such features must be deliberately designed and clearly explained to users to avoid patient safety risks and unnecessary burdens on health care professionals.

## Introduction

### Background

Online access to electronic health records (EHRs) has become a cornerstone of patient empowerment and transparency across Scandinavia. Patient-accessible EHRs (PAEHRs) allow patients to read their clinical notes, test results, and referrals through national patient portals [1] and give them the ability to identify errors in their EHRs [2-4]. However, the idea of patient contribution to the record has remained controversial.

Patient contribution to PAEHRs can take many forms. Health care systems internationally have explored patient-generated health data, secure messaging, and preconsultation surveys. In the United States, a study showed that allowing patients to provide input during the care process, such as designing the agenda before an appointment, led to perceived improvement in communication for over 70% of both patients and health care professionals (HCPs) [5]. Furthermore, 97% of US clinicians deemed patient-written agendas a good idea after a 12-month pilot intervention, and 70% usually or always included them in consultation notes [6]. However, research into direct patient-HCP messaging suggests that HCPs can find responding to patients’ messages tedious and overwhelming [7].

One of the most critical areas for patient contribution is in the realm of patient safety. Medical safety experts describe PAEHRs as a “transforming concept” [8], where more eyes on the record can prevent diagnostic delays and ensure timely follow-up of tests and referrals [9]. Crucially, patient-led error identification may allow clinicians to avoid relying on incorrect data, thereby reducing the risk of poor diagnostic or treatment decisions or clinical errors [10]. Despite this potential, the transition from passive reading to active reporting remains underexplored. In a US study of 22,000 patients, 1 in 5 reported finding an error, 40% of which were perceived as serious [11]. In Sweden, a survey found that approximately half of patients in mental health care had identified errors, compared with 35% in general care [12]. However, the lack of a digital reporting mechanism means patients wishing to have errors corrected must find an alternative method, such as discussing them during consultations or telephone calls. Consequently, many patients who identify errors take no action at all [12,13], a frustration reflected in a Finnish survey study in which the inability to correct errors was cited as a highly negative experience with PAEHRs [14].

Although patient contributions to EHRs are increasingly common internationally, most mechanisms have been designed for specific purposes, such as symptom reporting [15,16], patient-generated data [17], previsit agendas [5,6], and limited instances of structured error reporting [18]. Much less is known about how patients would use an open-ended, free-text feature to react directly to their EHR content. A better understanding of the potential of PAEHR interaction could illuminate ways in which PAEHRs can move beyond static archives to become dynamic tools for clinical communication.

### Aim and Research Questions

This study aimed to explore how a PAEHR comment function was used by Swedish patients in the Uppsala region between 2015 and 2021. Based on the study aim, 3 research questions were selected:

1. To what extent did patients make comments on their EHRs?
2. What was the content of patients’ comments?
3. What types of comments were made (eg, the comments’ relation to EHR content, sentiment, and topic)?

## Methods

A mixed methods design was used to analyze patient comments to clinical notes accessible through the national PAEHR service in Sweden.

### Setting

Sweden has universal, publicly funded health care that is available to all patients. Public health care is decentralized, with regions and municipalities being responsible for care delivery under national guidance. Digital health services are available nationally, primarily through the 1177.se patient portal, which offers both general health and care information, as well as personalized services upon patient authorization with an electronic ID. The 1177.se patient portal is widely adopted, with up to 240 million visits annually [19]. The national PAEHR service, 1177 Journalen, is hosted within the portal and was first implemented in 2012 and made available nationwide in 2017. The service is currently accessible by everyone aged 16 years or older. Guardians have access to their children’s records until they turn 13; individuals aged 13 to 15 years may apply for access on a case-by-case basis, although this is uncommon in practice [20]. In December 2021, a total of 1,600,092 citizens logged into their PAEHR [19]. While information availability differs across regions due to the decentralized care organization [21], clinical notes can be accessed from connected HCPs by patients in all regions. In addition to reading their EHRs, patients can conduct an array of health tasks via the platform, including, but not limited to, renewing prescriptions, sending messages to clinics, scheduling appointments, and requesting prolonged sick leave. Messages sent via the platform cannot be addressed to a specific HCP, and message threads are closed once the clinic has responded.

Between 2015 and 2021, a “Patient’s comments” function was available in all 21 Swedish regions, allowing patients to add comments to their clinical notes. The function was created in response to patients’ wishes to contribute to and communicate information to HCPs in their EHRs. Comments could contain up to 500 characters and had to be related to a specific note in the record. The explicit purpose of the function was not clear, but one suggestion was to note matters for follow-up during upcoming consultations. Patients could create, edit, and delete comments. To use the function, patients were required, at each login, to navigate to an administration page and activate it (see a screenshot in Figure 1). Once the function was activated, patients could return to the clinical notes in the PAEHR, select the note they wanted to comment on, and write their comment (see a screenshot in Figure 2). The Swedish original versions of the screenshots are found in Multimedia Appendix 1. Comments were associated with a specific note, and only 1 comment per note was possible. Comments were private by default and visible only to the patient; however, in 1 region (Uppsala), patients could choose to make the comments available to HCPs. If patients chose this option, HCPs were notified with a small symbol in the EHR. HCP-visible comments were available only in this region due to constraints of technical infrastructure. The Uppsala region, with approximately 400,000 inhabitants in 2021, accounted for about 3.8% of Sweden’s total population of 10.4 million [22]. As the national patient portal was updated over time, the technical infrastructure that supported the patient comment functionality became obsolete, and the functionality was eventually removed nationally at the end of 2021. It has not been reintroduced.

Legal procedures before implementation established that patient comments were not public information. If they were, HCPs would have been obligated to read and address them if needed. For example, if HCPs read about symptoms that required immediate medical attention, they would have had to attend to this according to the Swedish Patient Safety Law. However, because the comments were considered not to be public information, HCPs had no such obligation [23].

**Figure 1:**
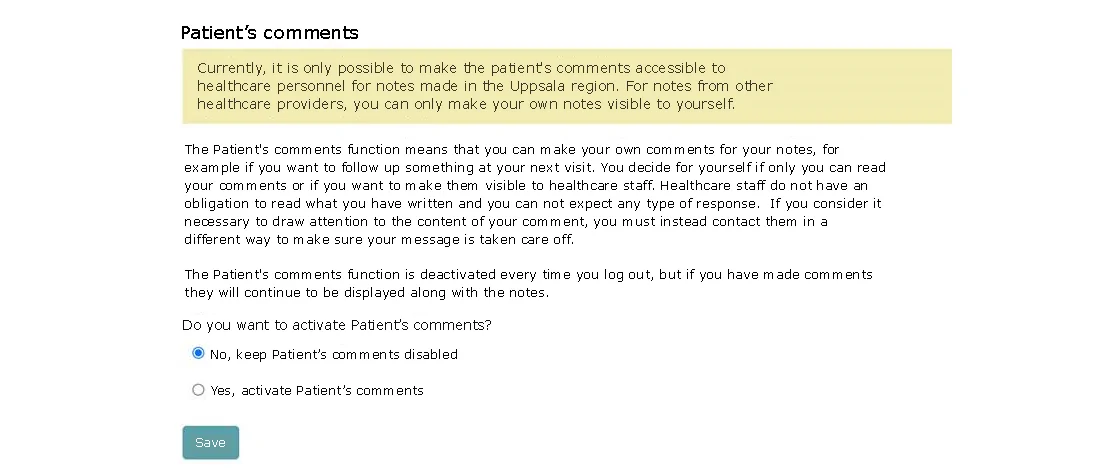
Screenshot showing how the comment function had to be activated on the patient portal (translated into English using Microsoft Copilot).

**Figure 2:**
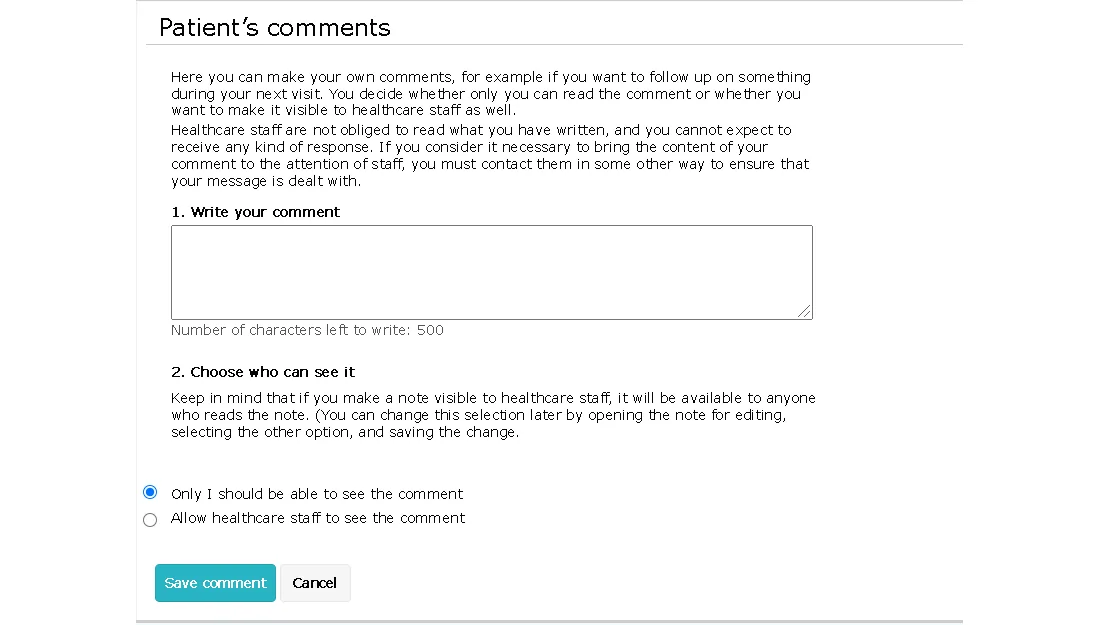
Screenshot of the comment function, showing the option to keep the comment private or share it with health care professionals (translated into English using Microsoft Copilot).

### Data Collection

National use statistics for the patient comment function were publicly available on the website of Inera AB, a company owned by Sweden’s regions and municipalities that manages the web platform of 1177 Journalen. For privacy reasons, only comments available to HCPs could be retrieved, and this was implemented only in the Uppsala region. The extracted data consisted of anonymized data with unique patient IDs (which could not be connected to the individuals and were used only to indicate which comments were written by the same person), comment content, and the date and time when comments were posted. We were unable to retrieve any additional information about the comment writers, such as age, gender, or diagnosis, or the actual clinical notes to which the comments were connected.

### Data Analysis

Comments were analyzed inductively using a thematic content analysis approach informed by the 6-step process of Braun and Clarke [24]. The authors first familiarized themselves with the data by reading through the comments. MH then analyzed 400 comments to develop a preliminary coding framework. Next, JH used this framework to analyze all 1670 comments. Coding and interpretations were discussed iteratively between the authors, and the framework was refined throughout the process. Sentiment (positive, negative, or neutral) was coded based on whether the comment expressed appreciation, criticism, or neither. In addition, comments were classified according to whether they referred to note content. Guided by our previous research, which found that patients with a history of mental health care have more negative experiences with their EHR, patients who referenced mental health care in at least 1 comment were tagged and analyzed separately to explore differences in comment volume and the proportion of negative sentiment cues [12,25]. Before analysis, content potentially identifying HCPs was deidentified by 1 of the authors. All quotes selected for manuscript inclusion underwent manual review to exclude identifiable information.

The qualitative analysis was conducted by JH and MH, 2 Swedish-speaking researchers with experience in health informatics and PAEHRs. Both have previously explored patients’ and HCPs’ perspectives on PAEHRs related to safety and documentation errors. Consequently, they entered the analysis with an awareness of potential patient perspectives on PAEHRs and care.

In addition to the qualitative analysis, descriptive statistics were used to summarize comment frequency, length, sentiment, and content categories. Proportions were reported with 95% CIs. Patients who referenced mental health care in at least 1 comment were compared with other patients regarding comment frequency and sentiment, with analyses conducted in SPSS (version 30.0; IBM Corp). The number of comments submitted per patient was summarized using medians because of the skewed distribution.

The qualitative and quantitative analyses were conducted in parallel and integrated at the interpretation stage. Quantitative findings on comment frequency, sentiment, and note-relatedness were used to contextualize and corroborate the qualitative categories, allowing patterns identified inductively to be examined against the broader data.

### Ethical Considerations

All procedures performed in this research were conducted in compliance with relevant national laws and institutional guidelines and were approved by the Swedish Ethical Review Authority (2022-02160-01). The privacy rights of all human participants were strictly observed. Because this study involved a retrospective analysis of information originally created for HCP review within the system, the requirement for informed consent was waived by the Swedish Ethical Review Authority. All data were processed in accordance with privacy regulations to ensure the confidentiality of the participants.

## Results

### Quantitative Findings

#### Summary

During the 6 years (2015-2021) that the comment function was available, patients in the Uppsala region left 1670 comments available for HCPs to view. Of these, 15 (0.9%) contained no meaningful content, such as random characters or the word “test.” The remaining 1655 (99.1%) were analyzed. The comments were produced by 659 patients, which equates to an average of 2.5 comments per patient (Table 1). These comments equate to 1.65 commenting patients per 1000 inhabitants in the Uppsala region. However, this may be a conservative estimate because the function required manual activation and was not widely advertised. All comments were written in Swedish, except for a few that were written in English. Nationwide, 5154 comments were created by patients and were available only to them. We do not know what these comments entailed.

As shown in Figure 3, patient comments in Region Uppsala were primarily directed to HCPs’ view across all years of implementation. Both categories had their maximum volumes in 2020 and 2021, with comments for own view peaking at 82 in 2021 and comments for HCPs at 336 in 2020.

**Table 1:** Characteristics of the patient comments.

Measures			Values
Patients, n			659
Comments, n			1670
Comments with meaningful content, n (%)			1655 (99.1)
Comments with irrelevant content, n (%)			15 (0.9)
**Comments per patient**			
	Minimum comments	1	
	Maximum comments	99	
	Median	2	
**Words per comment**			
	Minimum words in a comment, n	1	
	Maximum words in a comment, n	104	
	Average	48.36	
	SD	28.00	
	Median	48.00	
**Distribution of comments per patient, n (%)**			
	1-5	601 (91.2)	
	6-10	39 (5.9)	
	11-15	11 (1.7)	
	>15	8 (1.2)	

**Figure 3:**
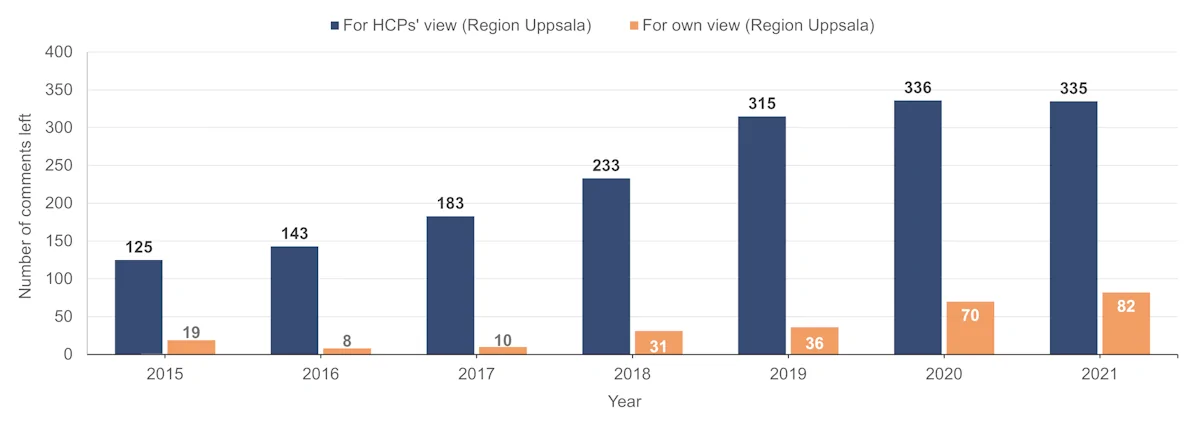
Number of comments left by patients for viewing by health care professionals (HCPs) and their own viewing in the Uppsala region between 2015 and 2021.

#### Reference to Note Content vs Other Matters

Of the 1655 comments with relevant content, the majority (948, 57.3%) concerned matters unrelated to note content, such as requesting contact, updating contact information, or complaining about a care encounter. Conversely, 42.6% (705/1655) directly pertained to the content of EHR notes. For 2 (0.1%) comments, it was unclear whether they referred to the note or other matters.

#### Sentiment Analysis

More than half of the comments expressed a neutral sentiment (920/1655, 55.6%). Of the remaining comments, 42.4% (702/1655) were negative. Negative comments often expressed dissatisfaction with care or disagreement with note content. For example, 1 patient expressed:

I think it’s upsetting that it says I’m someone who can’t make up my mind, is indecisive, and doesn’t have a realistic view of how I’m feeling. I’m very disappointed by this text and by the fact that [HCP] sees me this way.

ID 125698

Only 2% (33/1655) were positive.

#### Mental Health Care

A minority of users (74/659, 11.2%) referred to mental health care in at least 1 comment, yet they accounted for 29.9% (495/1655) of comments and wrote more comments (median 7 vs 2 comments per patient among those who did not mention mental health care). Furthermore, comments written by these patients were more commonly negative (313/495, 63.2%) than those written by patients who did not mention mental health care (389/1160, 33.5%; *P*<.001). One patient wrote the following:

There are so many mistakes in these notes from the psychiatric emergency room! I never said that I moved to [city] from [city] a year ago. I’m as far from being suicidal as you can get. I DO NOT HAVE AN EATING DISORDER!!

ID 869599

### Qualitative Categories

#### Summary

In total, 8 categories were developed through analysis of the comments. Two categories were highly prevalent (30%-50% of comments), 2 were moderately prevalent (20%-30% of comments), and 4 had low prevalence (<4% of comments). Figure 4 presents a visual display of the categories.

**Figure 4:**
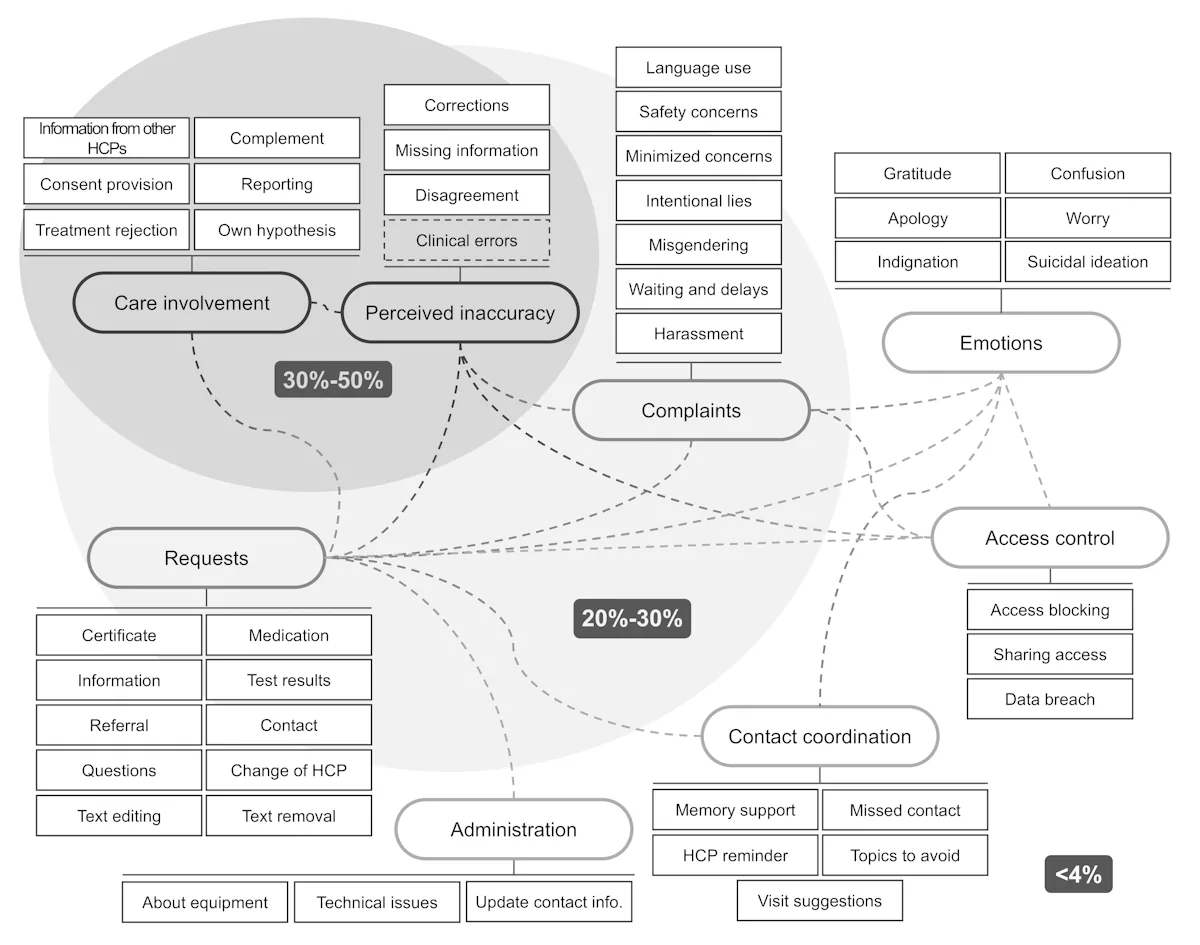
Visual display of the qualitative categories identified in patient comments. Darker shades of gray indicate higher frequency. Comments categorized as irrelevant (n=15) were not included. HCPs: health care professionals.

#### Care Engagement

Almost half of the comments (752/1655, 45%) demonstrated patient engagement in their own care, often through symptom updates or additional health information:

I’ve now taken antibiotics for 7 days, and the pain is practically gone.

ID 226

Noticed a weight loss of 10 kg during the recent weeks—checked my weight after the appointment.

ID 544

Patients also showed engagement by providing consent to procedures, conveying information from other HCPs, declining treatment, and offering their own hypotheses about their health conditions. Several patients even included links to external sources to support their interpretations.

#### Perceived Inaccuracies

Approximately one-third of comments (590/1655, 35.7%) referred to errors in, corrections to, missing information in, or disagreement with information encountered in the EHR. Perceived erroneous information was, in many cases, related to symptoms and current and past care, but often also included personal descriptors and other personal information. Commonly, patients described the error and how it should be corrected:

It’s the left foot, not the right as it says in the note.

ID 140

Perceptions of encounters that patients found inaccurate—such as “this didn’t happen”—tended to be related to complaints.

We didn’t agree that I should start with [medication name] because of side effects.

ID 117

While 590 comments identified errors in the EHR, there was an overlap with 245 comments that described clinical errors. Given this paper’s focus on documentation errors in the EHR, the clinical error data are reported in a different publication.

#### Complaints

About 1 in 4 (390/1655, 23.6%) comments contained complaints about a range of topics, including delayed care, negative encounters, harassment, misgendering, wording, feeling not taken seriously, and perceived lies from HCPs in notes. A number of the complaints contained threats to report HCPs. A few complaints were written using sarcasm.

Doctor was rude, stressed and didn’t listen to me.

ID 5

I have never been treated so badly by a doctor. She was cold and unsympathetic and belittled my pain as if I had come there and made it all up??

ID 469

#### Requests

One-fifth of comments (355/1655, 21.5%) concerned questions or some type of request from patients to HCPs. Requests were related to almost all other categories and concerned areas including error rectification, contact requests, medication, support, test results, and changes to or removal of text in a note.

I wish for cortison treatment and a check of insulin needs.

ID 96

Can you help me with medication until I have an appointment with you? I have severe difficulty falling asleep.

ID 452

Numerous patients reported experiences of pain and requested renewed or new prescriptions for various types of pain medications.

I need another type of pain medication as [current medication] does not take away my pain to any significant extent.

ID 11

#### Low-Frequency Categories

Among the less frequent categories, emotions were present in 3.6% (59/1655) of comments. For example, some patients conveyed gratitude to their HCPs for their support and care, while others were apologetic. Furthermore, several expressed worry about their health and confusion from not understanding or recognizing the note information. Other emotions were indignation, such as when feeling accused of misusing medication, and suicidal ideation. A similarly frequent category, accounting for 3.0% (50/1655) of comments, was related to patients coordinating care contacts and appointments with HCPs. Less than 1% pertained to access control (14/1655, 0.8%) or administration (7/1655, 0.4%).

### Integration of Quantitative and Qualitative Findings

Together, the quantitative and qualitative findings show how patients used the comment function as a broad communication channel rather than as a tool specific to the notes. The quantitative analysis showed that most comments (948/1655, 57.3%) were unrelated to note content, and this was reflected qualitatively in the prevalence of care engagement, requests, and complaints—categories that extended well beyond reactions to specific clinical notes. The sentiment analysis revealed that while most comments were neutral (920/1655, 55.6%), a notably high proportion were negative (702/1655, 42.4%), with only 2% (33/1655) being positive. This skew toward negativity was echoed qualitatively in the prevalence of complaints and perceived inaccuracies among the most common categories. These findings suggest that while the function held real value for a subset of motivated patients, the absence of clear guidance and a structured purpose shaped how it was used in ways that may not have aligned with its intended design.

## Discussion

### Summary

This study of a patient comment function implemented in the Swedish national PAEHR between 2015 and 2021 found that, in total, 659 patients left 1670 comments available to HCPs. Most comments were neutral, yet 2 in 5 comments were negative. It should be noted that the comment rate remained below 1 comment per 1000 inhabitants, indicating that the function was used by a small subset of patients. The most common categories were care engagement, perceived errors, complaints, and requests.

### Comparison With Existing Literature

The findings indicate that patients used the comment function as a general channel for communication about care-related needs, often with negative sentiment. Complaints about care encounters were common and align with previous Swedish research [26], and the particularly negative tone in mental health care echoes documented concerns among psychiatric HCPs [27]. Notably, over half of comments addressed matters unrelated to note content, suggesting that, without clear guidance, patients interpreted the open-ended function as a broad communication tool rather than a specific feedback feature.

The lack of clarity also led some patients to perceive the function as a direct communication channel with the HCP. While some patients acknowledged that HCPs were unlikely to read their comments, others used greeting phrases (eg, “Hello [HCP name]!”), anticipating a reader. When implemented, Swedish HCPs worried that the function could give patients a false impression of dialogue, potentially complicating clinical encounters [28]. Similar issues regarding patient expectations have been reported in studies about patient messages [7]. This disconnect between expectations and the legal framework raises important questions. When patients communicate urgent concerns, such as suicidal ideation, through a channel that is not reviewed by mandate, responsibility among patients, HCPs, and health care organizations becomes diffuse. These findings highlight the importance of clearly articulating the purpose of comment functions in PAEHRs, the necessity of enabling patients to request a response or action, and the value of considering whether such features require emergency protocols to prevent harm while maintaining a manageable HCP workload. Recent work also suggests potential value in using generative AI to support responses to patient portal messages [29].

The open-ended nature of the comment function differentiates it from structured feedback tools, such as a 9-question error-reporting form developed by Bell et al [30]. The Swedish comment function offered no defined procedures for management or response. It required patients to locate and activate it themselves, likely limiting its uptake. Despite this lack of structure, 97% of comments fell into 4 categories: care engagement, perceived errors, complaints, and requests. Similar patterns have been observed in patient-clinician emails in primary care [31], where requests (77%) and medical updates (19%) predominate. This concentration suggests a consistent set of patient communication needs that should inform future PAEHR designs. In addition, the categories indicate that patient contributions can extend beyond error reporting, allowing patients to raise issues that might otherwise go unaddressed. Although complaints are often rooted in negative experiences, they can provide valuable insight for preventing similar situations for other patients [32], yet they are also known to be emotionally taxing for HCPs and are not always viewed as opportunities for improvement [33]. Clearer guidance for patients on how to lodge complaints may therefore help ensure that such input is constructive and appropriately handled.

### Implications

Findings indicate substantial room for improvement in comment function design. Table 2 summarizes key issues and recommended strategies for future implementation. While patient contributions can support engagement, new interactive features must avoid increasing HCP workload—a documented concern related to patient messaging [7]. Further efforts should therefore balance patient benefits with strategies that minimize unnecessary burden on HCPs. The cessation of the function in 2021, despite evidence of meaningful patient use, illustrates how interactive features lacking clear governance structures may prove difficult to sustain in practice.

Current PAEHR implementations remain largely one-way, leaving opportunities for enhanced patient engagement, improved recall, and error identification only partially realized. Because HCPs are not notified when notes are read, they cannot use PAEHRs to communicate or verify information receipt, and their workload persists, with the added risk of negative patient reactions. Additional research is needed to explore how PAEHRs can generate benefits for HCPs. In this study, many patients attempted to share care updates, such as confirming receipt of information and sharing health metrics, which could support administrative or clinical workflows if properly integrated.

**Table 2:** Potential issues in the patient comment function and recommendations for future implementation.

Identified issue	Recommendations for improvement
Inadequate design	Ensure seamless function integration
The function was insufficiently integrated into the portal and required patients to navigate to a specific page to activate it, limiting visibility	Integrate the function directly into relevant portal views, minimize unnecessary navigation steps, and provide links to related information or alternative functions to support appropriate use
Lack of communication	Standardized guidance for users
Patients and HCPs^a^ lacked clear information about the existence, purpose, and intended use of the function	Clearly state the function’s intended use on the portal; encourage HCPs to discuss its use with patients, such as for follow-up information or confirmation of note review
Unclear function purpose	Define and clarify purpose
Patients were uncertain whether the function was intended for EHR^b^-related input, requests, or administrative matters, leading to inconsistent use	Distinguish EHR-related input from administrative requests and clearly indicate where to seek follow-up or submit complaints

^a^HCP: health care professional.

^b^EHR: electronic health record.

### Limitations

Several limitations should be noted. First, the absence of demographic data (eg, patient age and gender) due to privacy reasons precluded any analysis based on such factors. Second, we could not retrieve the original clinical notes, meaning that the relevance and accuracy of comments had to be inferred solely from the patients’ content. Third, the small sample size relative to the number of PAEHR users suggests a selection bias, as the function was not well publicized. The data may therefore represent the most motivated or frustrated users, rather than the general PAEHR population. In addition, no feedback from patients or HCPs was collected during implementation of the function or after its cessation in 2021, which limits our understanding of its significance. Finally, while we categorized errors, their clinical significance was not explored here and remains a subject for future analysis.

### Conclusions

In conclusion, our study of a PAEHR patient comment function indicated negative reactions from patients and unmet patient needs for communication. The findings suggest that future implementations of interactive features would benefit from a clearer distinction between note-related input and other patient communication. Efforts to enhance patient agency may contribute to improved documentation accuracy, care quality, and patient-clinician communication. However, these initiatives require carefully planned strategies and clear guidance to ensure patient safety and avoid imposing added strain on clinicians’ already limited time and resources.

## Appendix

Multimedia Appendix 1 Swedish original versions of the screenshots.

## Abbreviations

EHR: electronic health record
HCP: health care professional
PAEHR: patient-accessible electronic health record

## Data Availability

The data supporting the findings of this study are not available due to ethical restrictions related to participant anonymity and privacy. Access to the underlying data is therefore not permitted. Requests for further information regarding the data and the ethical restrictions may be directed to the corresponding author.
