# Supplementary material for: Patient Use of a Comment Function in Electronic Health Records: Mixed Methods Case Study
Source: J Med Internet Res. 2026 Oct 20;28:e98359. doi: 10.2196/98359 (PMC13732174; doi:10.2196/98359)
Supplement: Supplementary file 1 [file jmir_v28i1e98359_app1.pdf]

Appendix 1. Screenshots in Swedish.

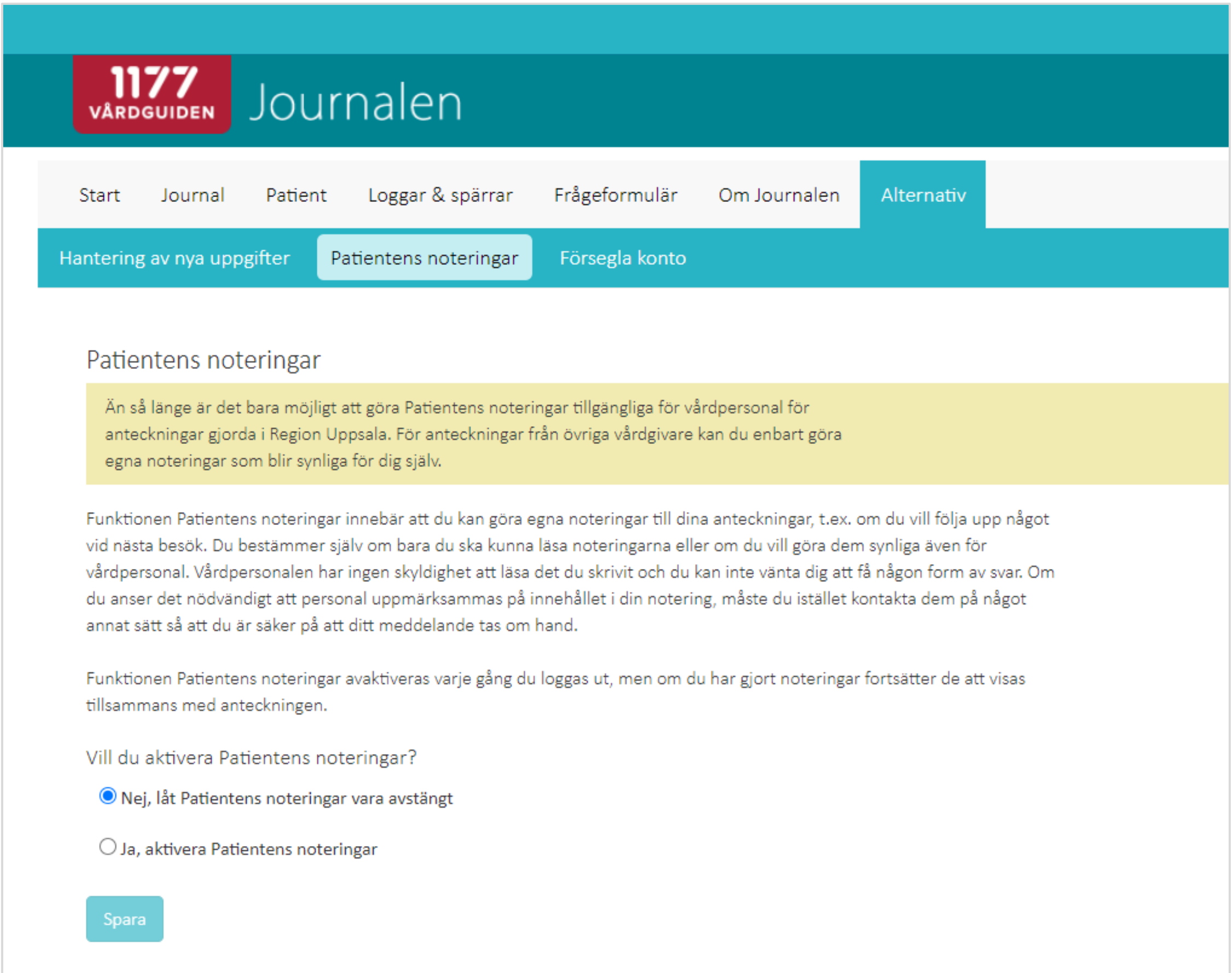

Fig A.1. Screenshots of how the comment function needed to be activated on the patient portal.

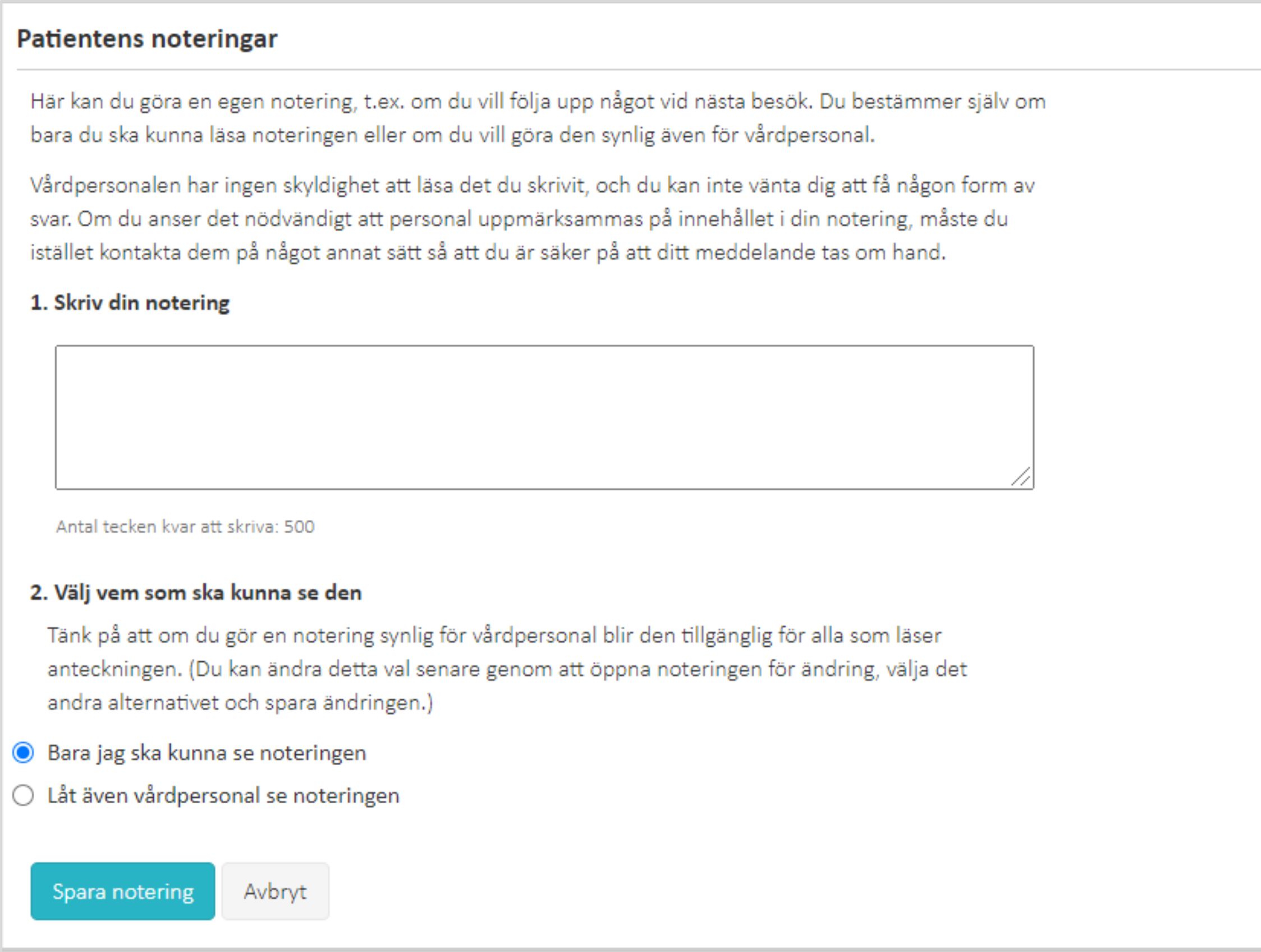

Patientens noteringar

Här kan du göra en egen notering, t.ex. om du vill följa upp något vid nästa besök. Du bestämmer själv om bara du ska kunna läsa noteringen eller om du vill göra den synlig även för vårdpersonal.

Vårdpersonalen har ingen skyldighet att läsa det du skrivit, och du kan inte vänta dig att få någon form av svar. Om du anser det nödvändigt att personal uppmärksammas på innehållet i din notering, måste du istället kontakta dem på något annat sätt så att du är säker på att ditt meddelande tas om hand.

1. Skriv din notering

Antal tecken kvar att skriva: 500

2. Välj vem som ska kunna se den

Tänk på att om du gör en notering synlig för vårdpersonal blir den tillgänglig för alla som läser anteckningen. (Du kan ändra detta val senare genom att öppna noteringen för ändring, välja det andra alternativet och spara ändringen.)

- (x) Bara jag ska kunna se noteringen
- ( ) Låt även vårdpersonal se noteringen

Spara notering | Avbryt

Fig A.2. Screenshot of the comment function, showing the choice to keep the comment private or to share it with healthcare professionals.

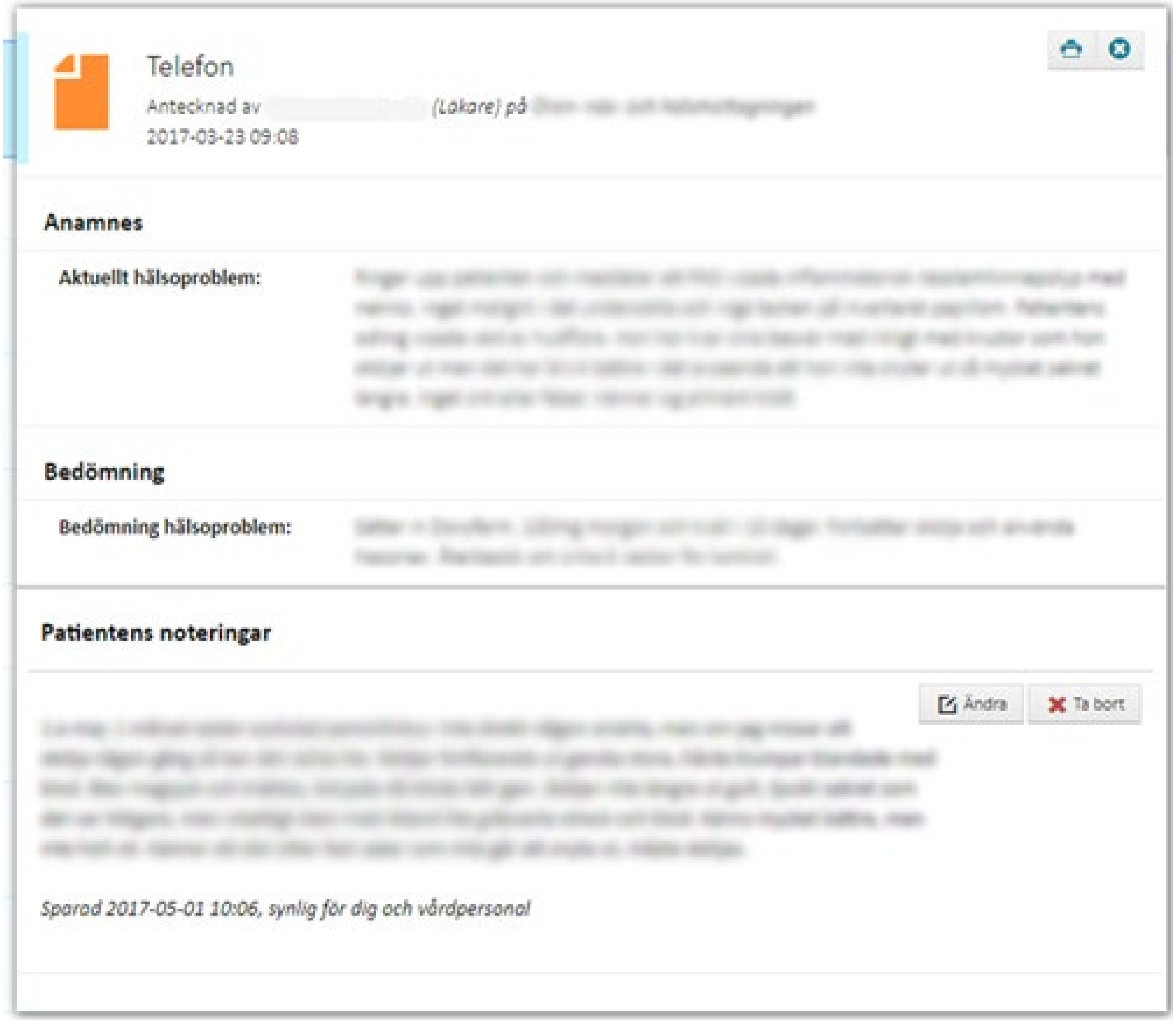

Fig A.3. Screenshot of how the comment looked in the electronic health record, located underneath the clinical note it was connected to.
